# Development and validation of the patient history COVID-19 (PH-Covid19) scoring system: a multivariable prediction model of death in Mexican patients with COVID-19

**DOI:** 10.1017/S0950268820002903

**Published:** 2020-11-26

**Authors:** J. Mancilla-Galindo, J. M. Vera-Zertuche, A. R. Navarro-Cruz, O. Segura-Badilla, G. Reyes-Velázquez, F. J. Tepepa-López, P. Aguilar-Alonso, J. de J. Vidal-Mayo, A. Kammar-García

**Affiliations:** 1 Unidad de Investigación UNAM-INC, Instituto Nacional de Cardiología Ignacio Chávez, Ciudad de México, Mexico; 2 Departamento de Endocrinología, Clínica de Obesidad, Instituto Nacional de Ciencias Médicas y Nutrición Salvador Zubirán, Ciudad de México, Mexico; 3 Facultad de Ciencias Químicas, Departamento de Bioquímica y Alimentos, Benemérita Universidad Autónoma de Puebla, Puebla, Mexico; 4 Facultad de Ciencias de la Salud y de los Alimentos, Departamento de Nutrición y Salud Pública, Programa UBB Saludable, Universidad del Bío-Bío, Chillán, Chile; 5 Departamento de Cardiología, Centro Médico Nacional La Raza, Instituto Mexicano del Seguro Social, Ciudad de México, Mexico; 6 Servicio de Endoscopia, Hospital Juárez Centro, Ciudad de México, Mexico; 7 Sección de Estudios de Posgrado e Investigación, Escuela Superior de Medicina, Instituto Politécnico Nacional, Ciudad de México, Mexico; 8 Departamento de Atención Institucional Continua y Urgencias, Instituto Nacional de Ciencias Médicas y Nutrición Salvador Zubirán, Ciudad de México, Mexico

**Keywords:** COVID-19, Mexico, prediction model, SARS-CoV-2, scoring system

## Abstract

Most of the existing prediction models for COVID-19 lack validation, are inadequately reported or are at high risk of bias, a reason which has led to discourage their use. Few existing models have the potential to be extensively used by healthcare providers in low-resource settings since many require laboratory and imaging predictors. Therefore, we sought to develop and validate a multivariable prediction model of death in Mexican patients with COVID-19, by using demographic and patient history predictors. We conducted a national retrospective cohort study in two different sets of patients from the Mexican COVID-19 Epidemiologic Surveillance Study. Patients with a positive reverse transcription-polymerase chain reaction for SARS-CoV-2 and complete unduplicated data were eligible. In total, 83 779 patients were included to develop the scoring system through a multivariable Cox regression model; 100 000, to validate the model. Eight predictors (age, sex, diabetes, chronic obstructive pulmonary disease, immunosuppression, hypertension, obesity and chronic kidney disease) were included in the scoring system called PH-Covid19 (range of values: −2 to 25 points). The predictive model has a discrimination of death of 0.8 (95% confidence interval (CI) 0.796–0.804). The PH-Covid19 scoring system was developed and validated in Mexican patients to aid clinicians to stratify patients with COVID-19 at risk of fatal outcomes, allowing for better and efficient use of resources.

## Introduction

The coronavirus disease (COVID-19) pandemic is the global phenomenon which is shaping modern societies in the year 2020, a reason why the severe acute respiratory coronavirus 2 (SARS-CoV-2) has been named the once-in-a-century pathogen that scientists and global leaders had been worrying for [1]. Although some countries were able to reach suppression (≤5 cases per million per day, and ≥20 tests per case) of the epidemic by August 2020, most others had persistent detection of new cases, with varying degrees of transmission and testing rates [2]. Non-pharmacological interventions may facilitate a country reaching control of disease spread; these interventions include: early lockdowns combined with other measures (school and workplace closures, social distancing, travel restrictions and restrictions on mass gathering and public events), and use of facemasks [3–6]. However, strict quarantines have an important social and economic impact, and countries such as Mexico have not been able to endure prolonged quarantines, resulting in a sustained transmission and death toll. Novel strategies such as switching between closures and keeping communities open [7], and regionalising closures in a timely manner [8] could provide useful in limiting the impact of COVID-19 in complex countries such as Mexico.

Mexico has been one of the most affected countries by COVID-19; disparate differences in patient outcomes have been noted be related to inequalities [9]. Low and middle-income countries often suffer from inadequate healthcare due to the lack of equipment, poor organisation, and scarce qualified healthcare professionals. Thus, what works in high-income countries may not work in low-income countries [10]. Therefore, there is a pressing need to develop accessible and simple tools to aid clinicians providing medical attention in the most unfavoured regions of Mexico.

Demographic and patient history risk factors for fatal outcomes in patients with COVID-19 have been characterised in large national cohorts [11–14], and broadly include: old age, sex (men), comorbidities, deprivation (a correlate of poverty) and belonging to certain ethnic groups. Other clinical, radiological and laboratory parameters at presentation have also been studied as risk factors for disease progression and death [14, 15]. Several diagnostic and prognostic models have been developed to be used in patients with COVID-19 [16]. However, most models include laboratory and radiographic variables which would be nearly impossible to collect in low-resource settings. Furthermore, these models have seldom been validated, are often inadequately reported or are overfitted due to a large predictor-to-outcome ratio [16–18], reasons that may limit their usefulness in real-world settings. Developing and validating models that only require demographic and patient history data, by using large national or multinational cohorts, may be a way to overcome these shortcomings to provide useful tools to clinicians in low-resource regions.

Therefore, we sought to develop and validate a multivariable prediction model of death in Mexican patients with COVID-19, by using demographic and patient history predictors.

## Method

### Study design

We conducted a national retrospective cohort study in two different sets of patients from the Mexican COVID-19 Epidemiological Surveillance Study [19] to develop and validate a multivariable prediction model of death in Mexican patients with COVID-19. Patient history variables were used as predictors of death as the outcome of interest. Blind assessment was not required since these are objective variables unlikely subjected to bias.

To develop the model, we included 264 026 patients studied between 28 February and 30 May 2020. All patients with a positive reverse transcription-polymerase chain reaction (RT-PCR) for SARS-CoV-2 were included to maximise the power and generalisability of results. Patients with incomplete data were excluded, whereas patients with the same demographic, clinical and follow-up variables were considered duplicated and only one entry was kept.

To validate the model, we included 592 160 patients studied between 1 June and 23 July 2020. Only patients with a positive RT-PCR for SARS-CoV-2 and complete unduplicated data were included to validate the model. We further performed simple random sampling of positive cases to increase statistical power in approximately 15% with respect to the sample used for developing the model.

### Source of data

Data are collected and regularly updated by the Mexican Secretariat of Health and are available in the Open Data platform of the Federal Government of Mexico [20]. A historical repository of individual datasets starting on 12 April 2020 is available through the General Directorate of Epidemiology [21]. Patients who met criteria of suspected COVID-19 case and were subsequently tested for SARS-CoV-2 were included in the study, starting on late February 2020 when the first suspected cases arrived in Mexico. Two diagnostic strategies are outlined in the National COVID-19 Epidemiological Surveillance Plan [19]: (1) testing of 10% of ambulatory patients with mild symptoms of respiratory disease and 100% of patients with respiratory distress at evaluation in one of the 475 monitoring units of viral respiratory disease (USMER, for its acronym in Spanish) which are strategically distributed to be representative of the Mexican population, and (2) testing 100% of patients who meet diagnostic criteria of Severe Acute Respiratory Infection (defined as shortness of breath, temperature ≥38 °C, cough and ≥1 of the following: chest pain, tachypnoea, or acute respiratory distress syndrome) who seek medical attention in non-USMER units.

Healthcare professionals collecting a diagnostic specimen are required to fill out a format containing demographic and patient history variables. Follow-up of all suspected COVID-19 cases is registered by accredited hospital epidemiologists (inpatients) and the responsible healthcare professional of every Local Health Jurisdiction (ambulatory patients), who ultimately upload data to the Respiratory Diseases Epidemiological Surveillance System. Results of diagnostic RT-PCR for SARS-CoV-2 are directly uploaded by the diagnostic facility; accreditation of diagnostic procedures by the Mexican Institute of Diagnostics and Epidemiological Reference is required to upload results. Reporting of deaths is obligatory and must be done in less than 48 h after occurrence. One caveat to this reporting method is that patients who are tested more than once in different jurisdictions may be duplicated. No variables that could lead to identification of patients are provided in datasets. Thus, the only way to eliminate duplications is through matching of cases with equal demographic and clinical variables. Specific information of treatments is not released.

Variables provided in the datasets are: origin (USMER, non-USMER), healthcare provider, state, birthplace, place of residency, nationality, indigenous language speaker, migratory status, type of medical attention (hospitalisation/ambulatory), admission date, symptom onset date, invasive mechanical ventilation (intubation (yes/no)), admission to intensive care unit (ICU) (yes/no), pneumonia (yes/no), date of death, contact with confirmed COVID-19 cases (yes/no), SARS-CoV-2 RT-PCR result (positive, negative, or pending), age, sex and current pregnancy, and the following comorbidities (yes/no): diabetes, hypertension, obesity, cardiovascular disease (CVD), chronic kidney disease (CKD), immunosuppression, asthma, chronic obstructive pulmonary disease (COPD) and smoking.

### Statistical analysis

Descriptive data were calculated and are provided as frequencies, percentages or mean with standard deviation (s.d.). Characteristics of patients in the model development and validation cohorts were compared through Student's *t*-test or *χ*^2^. A Cox regression model was applied to predict the risk of death. The risk of death was assessed through univariate analysis of the following variables: age, sex, current pregnancy, diabetes, COPD, asthma, immunosuppression, hypertension, CVD, obesity, CKD, smoking and time from symptom onset to medical attention. Age and time from symptom onset to medical attention were included in the model as quantitative variables, whereas the rest of the variables were modelled as dummy variables. Risk factors associated with death which were statistically significant (*P* < 0.05) were included to develop the multivariate regression model. All variables with a level of significance *P* < 0.1 were considered in the Cox regression model by using the Enter method. Variables that kept a level of significance *P* < 0.05 were used in the final model, which was evaluated through Harrell's *C*-statistic to determine its discrimination of death.

A scoring system was developed in accordance with the model proposed by Sullivan *et al*. [22]. Each risk factor was organised into categories and the reference value was determined as follows. Age was entered into the Cox regression model as a continuous variable and further categorised into 10 sets of years (<20, 20–29, 30–39, 40–49, 50–59, 60–69, 70–79, 80–89, 90–99 and >90); the midpoint between the nine values of each category was set as the reference value (*W_ij_*). The reference for age as a risk factor (*W_i_*_REF_) was set in the 20–29 years category since this group had the lowest mortality in a previous study performed in Mexican patients with COVID-19 [12]. For the rest of risk factors, the absence of comorbidities and being woman (sex) were set as the reference values. Each *W_i_*_REF_ was subtracted from *W_ij_* and multiplied by the regression coefficient (*β_i_*) of the risk factor to determine units of regression of distancing for every risk factor in the reference category *β_i_*(*W_ij_* − *W_i_*_REF_). A constant *B*, defined as the constant increase in units of risk for each 5-year increase, was obtained by multiplying *β_i_* × 5. Values for *B* were 0.25 in this study. Scores for each category were obtained with the equation *β_i_*(*W_ij_* − *W_i_*_REF_)/B. For every point in the risk score, the estimated risk of death (*p*) was calculated with the Cox proportional hazards regression analysis:
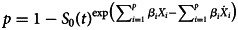
where *S*_0_(*t*) is the average survival according to mean values of every risk factor; 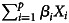 is substituted with each value of the risk score, times the *B* constant, plus the reference age value according to the *β_i_* of age and 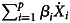 is the sum of every *β_i_* times the proportion or mean value of every risk factor.

To validate the model, we calculated the risk score for every patient and applied the Cox proportional hazards regression analysis. Estimated risks were obtained from both the scoring system and the observed risk in the regression analysis. The values obtained for patients in the validation cohort were distributed in percentiles (1–99) to determine the scoring categories. The estimated and observed risks were compared in each scoring category (percentiles 25, 50, 75 and 99).

The Kaplan–Meier analysis was performed to determine survival in each category of the scoring system, and a Cox regression analysis, to determine increases in the death risk for each category.

The association between the risk score and the probability of other adverse events (hospitalisation, invasive mechanical ventilation, pneumonia and admission to ICU) were also studied. The frequencies of each adverse event for every category in the scoring system were quantified and a binomial logistic regression analysis was performed to determine the risk of each adverse event according to the risk score; logarithms of the odds ratios (ORs) were graphed to establish the scoring value at which risk for every adverse event is increased.

All statistical analyses were performed using SPSS software v.21 and R statistical software v.3.6.2; figures were created in GraphPad Prism v.6. A value of *P* < 0.05 was used to establish statistical significance.

## Results

Out of 264 026 patients in the model development cohort, 84 627 had a positive RT-PCR for SARS-CoV-2, 140 553 were negative and 38 846 had pending results. After exclusion of patients with incomplete data and duplicated registries, 83 779 patients with a positive test were included to develop the model. Among the 592 160 patients in the model validation cohort, 256 488 patients had a positive result, 253 447 were negative and 82 225 had unreported results. After excluding duplicated and incomplete registries, and random sampling of positive cases, 100 000 patients were included to validate the model. Descriptive values of demographic characteristics, patient history and outcomes of patients (survivors and non-survivors) in both cohorts are provided in Table 1.

**Table 1.** Demographic characteristics, patient history data and outcomes in the model development and validation cohorts

	Development cohort, positive cases			Validation cohort, positive cases		
	Total *n* = 83 779	Survivors *n* = 7551	Non-survivors *n* = 9228	Total *n* = 100 000	Survivors *n* = 94 722	Non-survivors *n* = 5278
Sex						
Women, *n* (%)	36 393 (43.4)	33 370 (44.8)	3023 (32.8)^a^	48 129 (48.1)	46 304 (48.9)	1825 (34.6)^a^
Men, *n* (%)	47 386 (56.6)	41 181 (55.2)	6205 (67.2)^a^	51 871 (51.9)	48 418 (51.1)	3453 (65.4)^a^
Age, years	46.3 (15.9)	44.6 (15.4)	60 (14.2)^a^	43.9 (16.5)	42.9 (16.1)	61.3 (14.5)^a^
Smoking, *n* (%)	6966 (8.3)	6092 (8.2)	874 (9.5)^a^	6935 (6.9)	6538 (6.9)	397 (7.5)
Pregnancy, *n* (%)	537 (0.6)	523 (0.7)	14 (0.2)^a^	729 (0.7)	724 (0.8)	5 (0.1)^a^
Contact with confirmed case, *n* (%)	26 408 (31.3)	25 127 (33.7)	1100 (11.9)^a^	52 047 (52)	50 563 (53.4)	1484 (28.1)^a^
Comorbidities, *n* (%)						
Diabetes	14 735 (17.6)	11 235 (15.1)	3500 (37.9)^a^	14 036 (14)	12 010 (12.7)	2026 (38.4)^a^
COPD	1695 (2)	1183 (1.6)	512 (5.5)^a^	1244 (1.2)	1020 (1.1)	224 (4.2)^a^
Asthma	2466 (2.9)	2258 (3)	208 (2.3)^a^	2543 (2.5)	2427 (2.6)	116 (2.2)
Immunosuppression	1300 (1.6)	1020 (1.4)	280 (3)^a^	973 (1)	848 (0.9)	125 (2.4)^a^
Hypertension	17 609 (21)	13 696 (18.4)	3913 (42.4)^a^	17 162 (17.2)	14 970 (15.8)	2192 (41.5)^a^
CVD	2178 (2.6)	1640 (2.2)	538 (5.8)^a^	1875 (1.9)	1601 (1.7)	274 (5.2)^a^
Obesity	17 244 (20.6)	14 752 (19.8)	2492 (27)^a^	18 161 (18.2)	16 698 (17.6)	1463 (27.7)^a^
CKD	1944 (2.3)	1293 (1.7)	651 (7.1)^a^	1401 (1.4)	1131 (1.2)	270 (5.1)^a^
Time from symptom onset to medical attention, days	4.3 (3.2)	4.3 (3.2)	4.5 (3.1)^a^	4.3 (3.1)	4.2 (3.1)	5.1 (3.2)^a^
Hospitalisation, *n* (%)	29 535 (35.3)	21 257 (28.5)	8278 (89.7)^a^	19 316 (19.3)	14 753 (15.6)	4563 (86.5)^a^
Intubation, *n* (%)	2923 (3.5)	953 (1.3)	1970 (21.3)^a^	2260 (2.3)	778 (0.8)	1482 (28.1)^a^
Pneumonia, *n* (%)	22 785 (27.2)	15 644 (21)	7141 (77.4)^a^	16 742 (16.7)	12 122 (12.8)	4620 (87.5)^a^
ICU admission, *n* (%)	2791 (3.3)	1359 (1.8)	1432 (15.5)^a^	2383 (2.4)	1419 (1.5)	964 (18.3)^a^
Case-fatality rate (%)	11.02	–	–	5.3	–	–

Data are presented as mean values (s.d.), unless otherwise specified.

a Statistical significance with respect to survivors.

Variables included in the Cox regression model are presented in Table 2. Eight risk factors were included in the model (age, sex, diabetes, COPD, immunosuppression, hypertension, obesity and CKD); the only quantitative variable was age. Age, diabetes and CKD were associated with the greatest increases in death. The predictive model has a discrimination of 0.8 (95% confidence interval (CI) 0.796–0.804) and an average survival of 0.903 with the mean values for every risk factor. The patient history COVID-19 (PH-Covid19) scoring system assigns a score to every risk factor ultimately included in the predictive model (Table 3); the sum of scores for all risk factors included ranges from −2 to 25 points. Predicted probabilities of death in patients with a positive test for SARS-CoV-2 for every possible total value in the scoring system range from 0.74% to 99.82% (Table 4).

**Table 2.** Risk factors associated with death in Mexican patients with a positive diagnostic test for SARS-CoV-2 (model development cohort)

	Regression coefficient	Standard error	HR (95% CI)	*P* value	Mean or proportion
Age	0.050	0.001	1.05 (1.05–1.05)	<0.0001	46.27
Sex (men)	0.473	0.022	1.6 (1.54–1.68)	<0.0001	0.57
Diabetes	0.437	0.023	1.55 (1.48–1.62)	<0.0001	0.18
COPD	0.136	0.047	1.15 (1.05–1.26)	0.004	0.02
Immunosuppression	0.300	0.062	1.35 (1.19–1.52)	<0.0001	0.02
Hypertension	0.206	0.024	1.23 (1.17–1.29)	<0.0001	0.21
Obesity	0.337	0.024	1.4 (1.34–1.47)	<0.0001	0.21
CKD	0.620	0.042	1.86 (1.71–2.02)	<0.0001	0.02

HR, hazard ratio; 95% CI: 95% confidence interval; COPD, chronic obstructive pulmonary disease; CKD, chronic kidney disease.

**Table 3.** PH-Covid19 risk score to predict death in patients with COVID-19

Risk factor	Score
Age	
<20	−2
20–29	0
30–39	2
40–49	4
50–59	6
60–69	8
70–79	10
80–89	12
90–99	14
>99	15
Sex	
Male	2
Comorbidities	
Diabetes	2
COPD	1
Immunosuppression	1
Hypertension	1
Obesity	1
CKD	2

COPD, chronic obstructive pulmonary disease; CKD, chronic kidney disease.

Range of values: −2 to 25.

Reference categories for included variables: age (20–29 years), sex (woman), diabetes (no diabetes), COPD (no COPD), immunosuppression (no immunosuppression), hypertension (no hypertension), obesity (no obesity), CKD (no CKD).

**Table 4.** Estimated risk of death according to every possible score in the PH-Covid19 score, in Mexican patients with a positive test for SARS-CoV-2

Total value	Estimated risk (%)	Total value	Estimated risk (%)
−2	0.74	12	21.72
−1	0.94	13	26.98
0	1.21	14	33.22
1	1.55	15	40.45
2	1.99	16	48.60
3	2.55	17	57.46
4	3.26	18	66.63
5	4.17	19	75.56
6	5.32	20	83.62
7	6.77	21	90.20
8	8.61	22	94.94
9	10.92	23	97.83
10	13.80	24	99.27
11	17.36	25	99.82

**Fig. 1.**
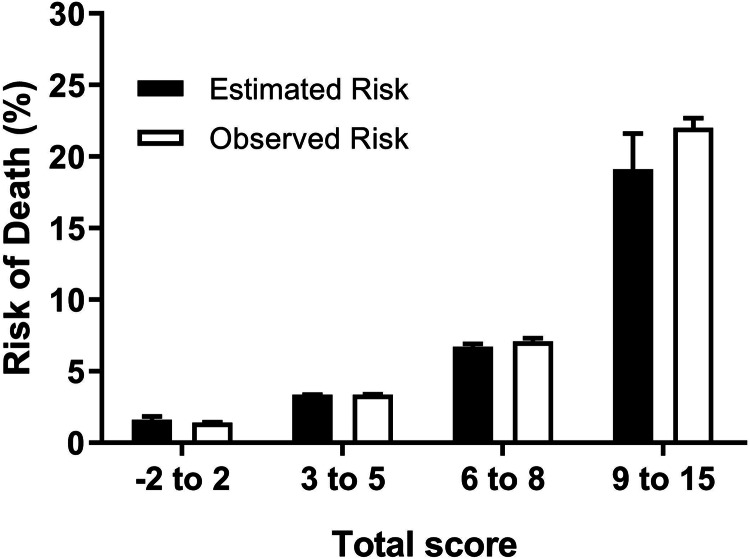
Estimated and observed risks of death in Mexican patients with a positive test for SARS-CoV-2, according to categories derived from the PH-Covid19 scoring system. Data are presented as mean and 95% CIs.

Baseline characteristics and outcomes of patients in the validation cohort were statistically significantly different from those in the model development cohort, except for time from symptom onset to medical attention (Table 1). Results of the Cox regression model applied to the validation cohort are provided in Supplementary Table S1. Calculated scores in the validation cohort reflected the following distribution according to percentiles: −2 to 2 points, percentile 1 to 25; 3 to 5 points, percentile 25–50; 6 to 8 points, percentile 51 to 75 and 9 to 15 points, percentile 76 to 99. Patients >99 percentile were considered as extreme values. Estimated risks and the observed risks of death obtained from the Cox proportional hazard regression analysis, in relation to the scores in each percentile, are presented in Figure 1. The estimated and observed risks were similar for every group (−2 to 2 points, 3 to 5 points, 6 to 8 points, and 9 to 15 points) and were strongly correlated (*r* = 0.98, *R*^2^ = 0.96, *P* < 0.0001).

**Fig. 2.**
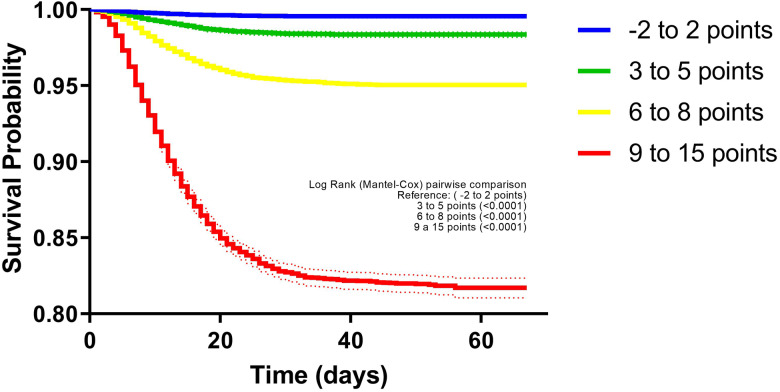
Kaplan–Meier survival curves in Mexican patients with a positive test for SARS-CoV-2, according to categories derived from the PH-Covid19 scoring system. Dashed lines represent 95% CIs.

In the survival comparison between the groups (−2 to 2 points, 3 to 5 points, 6 to 8 points, and 9 to 15 points) generated after the percentile distribution (Fig. 2), survival was lower with increasing scores; survival in the −2 to 2 points group was 99.6%; in the 3 to 5 points group, 98.6%; in the 6 to 8 points group, 95.7%, and in the 9 to 15 points group, 84.3%. Groups were compared against the −2 to 2 points group (*P* < 0.0001 for all comparisons). In the Cox regression analysis, increased risk of death occurred in patients in the 3 to 5 points group (hazard ratio (HR): 3.54, 95% CI 2.85–4.39), 6 to 8 points group (HR: 10.67, 95% CI 8.78–12.98), 9 to 15 points group (HR: 41.9, 95% CI 34.7–50.7) and >15 points group (HR: 87.6, 95% CI 70.1–109.5) compared with the −2 to 2 points group (reference category).

In accordance with this, risk categories were derived from the scoring system: low (−2 to 2 points), medium-low (3 to 5 points), medium (6 to 8 points), medium-high (9 to 15 points), and high (>15 points).

**Fig. 3.**
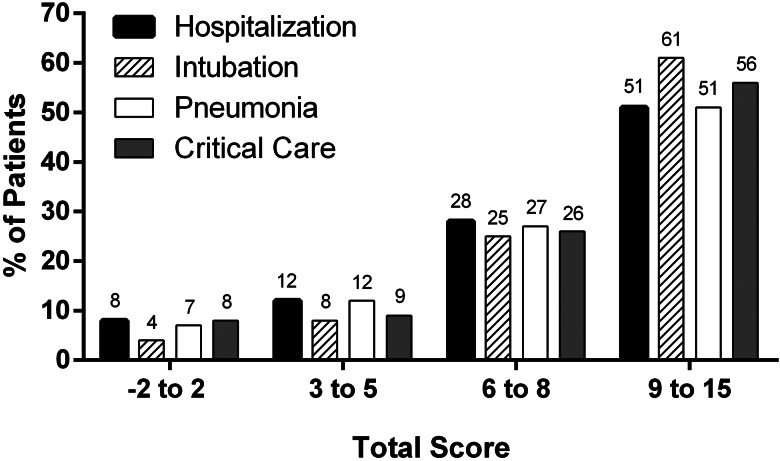
Prevalence of adverse events in Mexican patients with a positive test for SARS-CoV-2, according to categories derived from the PH-Covid19 scoring system.

The prevalence for every adverse event in each category of the scoring system are provided in Figure 3; results of the logistic regression analysis to determine the risk of adverse events for each risk category in the PH-Covid19 scoring system are given in Supplementary Table S2. Tendencies of risk increment for each adverse event with augmenting scores (Supplementary Fig. S1) reflect that risk for any adverse event starts at a value of 5 points.

## Discussion

We have developed and validated the PH-Covid19 score, a multivariable prediction model of death in Mexican patients with COVID-19, by using different datasets from the Mexican COVID-19 Epidemiological Surveillance Study. This scoring system has been created to aid clinicians working under resource-strained conditions to early stratify patients with COVID-19 according to their risk of fatal outcomes, without the need to perform laboratory or imaging studies.

Sex (men), which increases 2 points in our score, was correlated with death in our study and others [11, 12, 14, 15, 23]. Similar to other studies, we found that older age is the main risk factor for dying from COVID-19, with every 10-year increase associated with the largest increases in the HR [11, 14, 23]. In a recent prospective validation study of prognostic models for COVID-19, none of the models that predicted mortality in patients with COVID-19 were better than age alone to predict in-hospital mortality [24]. This may be explained by the fact that many models for COVID-19 have failed to account for the large increases in mortality risk due to increasing ages. In the PH-Covid19 scoring system, 10-year increases add 2 points, starting from 30 years, whereas being <20 years subtracts 2 points.

Diabetes and CKD resulted in 2-point increases in the score. Other studies had similar findings to ours [12, 13, 15, 23]; one study found an uncertain increased risk attributable to diabetes (HR: 1.14, 95% CI 0.91–1.43) in critically-ill patients [14]; in another study, uncontrolled diabetes further increased the risk (HR: 1.95, 95% CI 1.83–2.08) with respect to controlled diabetes (HR: 1.31, 95% CI 1.24–1.37), whereas CKD stages 4–5 caused a greater increase (HR: 2.52, 95% CI 2.33–2.72) compared to CKD stages 3a–3b (HR: 2.52, 95% CI 2.33–2.72) [11]. Obesity had the third strongest association with death, resulting in a 1-point increase in our scoring system; other studies have had similar findings [12, 23]. Adjusted risk of death by obesity occurred with gradual increases in higher obesity classes [11] and no evident or clear risk was identified in another study for class I and II obesity, whereas class III obesity was significantly correlated with death [14]. This last study, however, included only patients admitted to ICUs who had a high prevalence of comorbidities, which are independent risk factors for ICU admission [12].

Hypertension was a risk factor for death similar to other studies [12, 13], and resulted in a 1-point increase in the scoring system. In one large study, risk of death for hypertension adjusted by all covariates apparently reduced the risk (HR: 0.89, 95% CI 0.85–0.93), likely reflecting an artificial reduction of risk driven by diabetes and obesity since age and sex adjusted risk increased the risk (HR: 1.09, 95% CI 1.05–1.14) [11]. COPD also increased 1 point in our model, being also associated with death in other studies [11–13, 23].

Results from our validation did not provide different patient history predictors which could enhance the performance of our model. Thus, model updating was not required. The robustness of the model is reflected in the standard errors and CIs that imply an adequate estimation of population parameters. Furthermore, standard error of the regression coefficient indicates the dispersion of this statistic for the studied population, and the CIs calculated for every risk factor allow us to interpret that the estimations of HRs are precise. Similarly, comparisons of predicted and observed risks for every score groups are not different since their CIs overlap. Survival analyses show that even though survival rates for contiguous risk categories are close to each other, their CIs are equally close to each estimated survival probability, thereby supporting a robust estimation of survival according to risk categories in our study.

Some of our findings raise concerns regarding quality and access to healthcare in Mexico. 10.3–13.5% of patients who died in our cohorts did not receive in-hospital care at any moment of the disease, only 15.5–18.3% of patients were admitted to an ICU before dying and only 21.3–28.1% of patients who died were intubated; conversely, intubation in survivors was unusually low (0.8–1.3%). In other studies of hospitalised-only patients, 53–72% of non-survivors were admitted to an ICU and 51–59% received invasive mechanical ventilation [15, 25, 26]. Observed mortality in COVID-19 patients under 60 years is lower when access to healthcare is not a limitation [27]; non-survivors in our cohorts were younger (mean age 60–61.4 years) than those in other studies (67–80 years) [14, 15, 23, 28].

Patients diagnosed with pneumonia in both cohorts were 12.8–21% among survivors and 77.4–87.5% of non-survivors. These numbers are low compared to the prevalence of chest computed tomography-scan abnormalities which occur in 67.3–70.8% of asymptomatic/pre-symptomatic patients [29, 30], 95.5% of patients with mild COVID-19 [31] and 98% of all COVID-19 patients included in a meta-analysis [32]. Chest X-ray, on the other hand, may be normal in up to 63% of patients with early COVID-19 pneumonia [33]. Nonetheless, the low proportion of pneumonia in non-survivors suggests non-optimal diagnosis of pneumonia may be occurring in Mexico. The lack of an operational definition may have contributed since clinicians could have defined pneumonia differently based on clinical and/or radiographical findings. Other possibilities should be explored, including knowledge of Mexican clinicians on how to diagnose pneumonia and access to radiological studies during the pandemic in low-resource settings.

One recent model developed in a large cohort of in-hospital patients from a large cohort in the UK accurately predicts in-hospital death (area under the curve (AUC): 0.79, 95% CI 0.78–0.79) [34]. However, the 4C mortality score may be limited due to not accounting for the large increases in risk of death for every 10-year age category. This scoring system is easy to use, however, it requires input of two laboratory values (C-reactive protein and urea) which may limit its use in low-resource contexts and, differently to ours, its use is limited to in-hospital patients.

Three prognostic COVID-19 models have been developed in Mexican patients. The LOW-HARM model [35] is a 100-point scoring system calculated by inputting patient history and laboratory values, in which 65 points was set as the cut-off value to predict death (AUC: 0.80, 95% CI 0.77–0.84), similar to the PH-Covid19 scoring system (AUC: 0.80, 95% CI 0.796–0.804) which advantageously only requires patient history predictors. Another scoring system uses age (cut-off 65 years), comorbidities and pneumonia to predict death [36]. This model was accurate at predicting death and other adverse events but has the limitation of not accounting for the large increases in risk for every 10-year category or similar. Also, the model by Bello-Chavolla *et al*. was developed using one dataset of the Mexican Epidemiological Surveillance Study, which unfortunately had no operational definition for pneumonia as discussed earlier. A third model was developed and validated to predict the risk of admission to ICU; the ABC-GOALS model was developed in three versions: clinical, clinical + laboratory and clinical + laboratory + imaging predictors [37].

In one systematic review of existing prediction models for diagnosis and prognosis of COVID-19, the use of any of the reviewed models was discouraged since, out of 91 diagnostic and 50 prognostic models, all were at high risk of bias due to methodological constraints and poor reporting [16]. Predictive models of death (eight) often excluded patients that had not developed the outcome of interest, did not account for censorship, inadequately reported discrimination and calibration of the model, and had a high risk of bias according to PROBAST evaluation, despite authors claiming good global performances of their models. We have addressed these concerns in our development and validation of the PH-Covid19 scoring system.

One strength of our model is that it was developed and validated in cohorts including both ambulatory and hospitalised patients, whereas most other prognostic models for COVID-19 have been developed in hospitalised-only patients.

Another strength of our study is that we were able to perform a type 3 analysis according to TRIPOD by using individual datasets to develop and validate our model; this design allows for external validation of the performance of a model [38]. It is worth highlighting that sample sizes in both cohorts include thousands of patients, which adds robustness to our model.

One limitation of our study is that certain diseases (cancer, haematological malignancies and neurologic diseases) and specific states of a disease (obesity class, former or current smoker and control of diabetes, hypertension and asthma) which increase the risk of dying from COVID-19 [11] were not studied since they are not provided in the datasets. Furthermore, we were not able to study other social determinants and population factors which could be having an important impact in patient outcomes [39, 40]. However, our model accounts for the main risk factors associated with death in patients with COVID-19, and not requiring inputting specific disease states makes it easier to be used by clinicians while minimising the risk of not having enough data to use the score with precision. Another limitation is that the epidemiological surveillance strategy in Mexico allows testing of only 10% of ambulatory patients. Furthermore, the operational definition of suspected COVID-19 case used in Mexico until 24 August 2020 had a low sensitivity (58.2%), but a high specificity (63.7%) compared to that used by the CDC (85.8% and 25.8%, respectively) [41]. Altogether, this means that our cohorts may include very few patients with asymptomatic COVID-19 and fewer patients with mild COVID-19 compared to other national datasets with higher testing rates. Since it was not possible to determine the exact number of patients with mild disease in our cohorts, we can only indirectly suggest that mild-disease patients could comprise around 71.5% and 84.4% of patients according to the fraction of non-hospitalised patients who survived, a number high enough to permit the use of this score in patients with mild-to-severe COVID-19.

Future models that could outperform ours in low-resource settings should account for the large increases in risk due to age, evaluate more comorbidities and disease states, avoid difficult-to-obtain laboratory and imaging parameters and evaluate the impact of social determinants of mortality in COVID-19. Also, it would be ideal for these models to come from large national or multinational cohorts including both ambulatory and hospitalised patients, similar to that of the OpenSAFELY study [42].

The PH-Covid19 score was created to be used in limited-resource settings where access to laboratory and imaging studies may be restricted. In places where this is not a limitation and for patients who are likely to have already been admitted to hospital (critical patients), other prognostic models may have a better performance than ours. However, clinicians should consider that most models have not been validated before deciding to use any COVID-19 diagnostic or prognostic model.

The PH-Covid19 score uses patient history predictors which are frequently known at the first contact with a patient, or can be interrogated rapidly, to predict death in patients with COVID-19. This score was developed and validated in Mexican patients to be used in low-resource settings where obtaining laboratory and radiographic studies may not be immediately possible. This score will aid clinicians to stratify patients with COVID-19 at risk of fatal outcomes to use healthcare resources more efficiently.

## Data Availability

The data that support the findings of this study are openly available in Historical COVID-19 Datasets of the Directorate General of Epidemiology of Mexico at https://www.gob.mx/salud/documentos/datos-abiertos-bases-historicas-direccion-general-de-epidemiologia [21].

## Supplementary material

For supplementary material accompanying this paper visit https://doi.org/10.1017/S0950268820002903. click here to view supplementary material

## References

[1] Gates B (2020) Responding to Covid-19 – a once-in-a-century pandemic? New England Journal of Medicine 382, 1677–1679. 10.1056/NEJMp2003762 32109012

[2] Lancet COVID-19 Commissioners, Task Force Chairs, and Commission Secretariat (2020) Lancet COVID-19 commission statement on the occasion of the 75th session of the UN general assembly. The Lancet 396, 1102–1124. 10.1016/S0140-6736(20)31927-9 PMC7489891 32941825

[3] Islam N (2020) Physical distancing interventions and incidence of coronavirus disease 2019: natural experiment in 149 countries. The BMJ 370, m2743. 32669358 10.1136/bmj.m2743 PMC7360923

[4] Lavezzo E (2020) Suppression of a SARS-CoV-2 outbreak in the Italian municipality of Vo’. Nature 584, 425–429. 32604404 10.1038/s41586-020-2488-1 PMC7618354

[5] Nussbaumer-Streit B (2020) Quarantine alone or in combination with other public health measures to control COVID-19: a rapid review. Cochrane Database of Systematic Reviews 4, CD013574. 10.1002/14651858.CD013574 PMC7141753 32267544

[6] Chu DK (2020) Physical distancing, face masks, and eye protection to prevent person-to-person transmission of SARS-CoV-2 and COVID-19: a systematic review and meta-analysis. The Lancet 395, 1973–1987. 10.1016/S0140-6736(20)31142-9 PMC7263814 32497510

[7] Cheong KH, Wen T and Lai JW (2020) Relieving cost of epidemic by Parrondo's paradox: a COVID-19 case study. Advanced Science 2002324, 1–8. 10.1002/advs.202002324 PMC7740105 33344130

[8] Siegenfeld AF, Taleb NN and Bar-Yam Y (2020) Opinion: what models can and cannot tell us about COVID-19. Proceedings of the National Academy of Sciences 117, 16092–16095. 10.1073/pnas.2011542117 PMC7368306 32581126

[9] Gutierrez JP and Bertozzi SM (2020) Non-communicable diseases and inequalities increase risk of death among COVID-19 patients in Mexico. PLoS ONE 15, e0240394. 33031467 10.1371/journal.pone.0240394 PMC7544063

[10] Roder-DeWan S (2020) Health system quality in the time of COVID-19. The Lancet Global Health 8, e738–e739. 32389194 10.1016/S2214-109X(20)30223-0 PMC7202854

[11] Williamson EJ (2020) Factors associated with COVID-19-related death using OpenSAFELY. Nature 584, 430–436. 32640463 10.1038/s41586-020-2521-4 PMC7611074

[12] Kammar-García A (2020) Impact of comorbidities in Mexican SARS-CoV-2-positive patients: a retrospective analysis in a national cohort. Revista de Investigacion Clinica 72, 151–158. 32584330 10.24875/RIC.20000207

[13] Guan W (2020) Comorbidity and its impact on 1590 patients with COVID-19 in China: a nationwide analysis. European Respiratory Journal 55, 2000547. 10.1183/13993003.00547-2020 PMC7098485 32217650

[14] Gupta S (2020) Factors associated with death in critically Ill patients with coronavirus disease 2019 in the US. JAMA Internal Medicine 02115, 1–11. 10.1001/jamainternmed.2020.3596 PMC7364338 32667668

[15] Zhou F (2020) Clinical course and risk factors for mortality of adult inpatients with COVID-19 in Wuhan, China: a retrospective cohort study. The Lancet 395, 1054–1062. 10.1016/S0140-6736(20)30566-3 PMC7270627 32171076

[16] Wynants L (2020) Prediction models for diagnosis and prognosis of COVID-19: systematic review and critical appraisal. The BMJ 369, m1328. 32265220 10.1136/bmj.m1328 PMC7222643

[17] Collins GS, van Smeden M and Riley RD (2020) COVID-19 prediction models should adhere to methodological and reporting standards. European Respiratory Journal 56, 2002643. 10.1183/13993003.02643-2020 PMC7377211 32703773

[18] Hooli S and King C (2020) Generalizability of coronavirus disease 2019 (COVID-19) clinical prediction models. Clinical Infectious Diseases 71, 897–897. 10.1093/cid/ciaa417 PMC7184363 32271865

[19] Directorate General of Epidemiology MEX (2020) Standardized Guideline for Epidemiologic and Laboratory Surveillance of viral respiratory diseases, May 2020 [Spanish]. Mexican Secretariat of Health. Mexico City, pp. 1–80. Available at https://www.gob.mx/cms/uploads/attachment/file/552972/Lineamiento_VE_y_Lab_Enf_Viral_20.05.20.pdf (Accessed 17 August 2020).

[20] Health Ministry MEX (2020) Information regarding COVID-19 cases in Mexico [Spanish]. Datos Abiertos 2020. Available at https://datos.gob.mx/busca/dataset/informacion-referente-a-casos-covid-19-en-mexico (Accessed 17 August 2020).

[21] Directorate General of Epidemiology MEX (2020) Historical COVID-19 Datasets [Spanish]. Datos Abiertos – Bases Históricas. Available at https://www.gob.mx/salud/documentos/datos-abiertos-bases-historicas-direccion-general-de-epidemiologia (Accessed 17 August 2020).

[22] Sullivan LM, Massaro JM and D'Agostino RB (2004) Presentation of multivariate data for clinical use: the Framingham study risk score functions. Statistics in Medicine 23, 1631–1660. 15122742 10.1002/sim.1742

[23] Docherty AB (2020) Features of 20 133 UK patients in hospital with covid-19 using the ISARIC WHO clinical characterisation protocol: prospective observational cohort study. The BMJ 369, m1985. 32444460 10.1136/bmj.m1985 PMC7243036

[24] Gupta RK (2020) Systematic evaluation and external validation of 22 prognostic models among hospitalised adults with COVID-19: an observational cohort study. European Respiratory Journal. Published online: 25 September 2020. doi: 10.1183/13993003.03498-2020. PMC7518075 32978307

[25] Richardson S (2020) Presenting characteristics, comorbidities, and outcomes among 5700 patients hospitalized with COVID-19 in the New York city area. JAMA 323, 2052. 32320003 10.1001/jama.2020.6775 PMC7177629

[26] Yang X (2020) Clinical course and outcomes of critically ill patients with SARS-CoV-2 pneumonia in Wuhan, China: a single-centered, retrospective, observational study. The Lancet Respiratory Medicine 8, 475–481. 32105632 10.1016/S2213-2600(20)30079-5 PMC7102538

[27] Karagiannidis C (2020) Case characteristics, resource use, and outcomes of 10 021 patients with COVID-19 admitted to 920 German hospitals: an observational study. The Lancet Respiratory Medicine 8, 853–862. 32735842 10.1016/S2213-2600(20)30316-7 PMC7386882

[28] Wu C (2020) Risk factors associated with acute respiratory distress syndrome and death in patients with coronavirus disease 2019 Pneumonia in Wuhan, China. JAMA Internal Medicine 180, 934. 32167524 10.1001/jamainternmed.2020.0994 PMC7070509

[29] Hu Z (2020) Clinical characteristics of 24 asymptomatic infections with COVID-19 screened among close contacts in Nanjing, China. Science China Life Sciences 63, 706–711. 32146694 10.1007/s11427-020-1661-4 PMC7088568

[30] Wang Y (2020) Clinical outcomes in 55 patients with severe acute respiratory syndrome coronavirus 2 who were asymptomatic at hospital admission in Shenzhen, China. The Journal of Infectious Diseases 221, 1770–1774. 32179910 10.1093/infdis/jiaa119 PMC7184401

[31] Liang T (2020) Evolution of CT findings in patients with mild COVID-19 pneumonia. European Radiology 30, 4865–4873. 32291502 10.1007/s00330-020-06823-8 PMC7156291

[32] Awulachew E (2020) Computed tomography (CT) imaging features of patients with COVID-19: systematic review and meta-analysis. Radiology Research and Practice 2020, 1023506. 32733706 10.1155/2020/1023506 PMC7378588

[33] Cleverley J, Piper J and Jones MM (2020) The role of chest radiography in confirming covid-19 pneumonia. The BMJ 370, m2426. 32675083 10.1136/bmj.m2426

[34] Knight SR (2020) Risk stratification of patients admitted to hospital with covid-19 using the ISARIC WHO clinical characterisation protocol: development and validation of the 4C mortality score. The BMJ 370, m3339. 32907855 10.1136/bmj.m3339 PMC7116472

[35] Soto-Mota A (2020) The low-harm score for predicting mortality in patients diagnosed with COVID-19: a multicentric validation study. Journal of the American College of Emergency Physicians Open. Published online: 15 October 2020. doi: 10.1002/emp2.12259. 10.1002/emp2.12259 PMC7675373 33230506

[36] Bello-Chavolla OY (2020) Predicting mortality Due to SARS-CoV-2: a mechanistic score relating obesity and diabetes to COVID-19 outcomes in Mexico. The Journal of Clinical Endocrinology & Metabolism 105, dgaa346. 32474598 10.1210/clinem/dgaa346 PMC7313944

[37] Mejía-Vilet JM (2020) A risk score to predict admission to the intensive care unit in patients with COVID-19: the ABC-GOALS score. Salud Publica de Mexico. Published online: 10 October 2020. doi: 10.21149/11684. 33021362

[38] Moons KGM (2015) Transparent Reporting of a multivariable prediction model for Individual Prognosis Or Diagnosis (TRIPOD): explanation and elaboration. Annals of Internal Medicine 162, W1–W73. 25560730 10.7326/M14-0698

[39] Mathur R (2020) Ethnic differences in COVID-19 infection, hospitalisation, and mortality: an OpenSAFELY analysis of 17 million adults in England. medRxiv. Published online: 23 September 2020. doi: 10.1101/2020.09.22.20198754.

[40] Cheong KH and Jones MC (2020) Introducing the 21st century's new four horsemen of the coronapocalypse. BioEssays 42, e2000063. 32227642 10.1002/bies.202000063

[41] Instituto Mexicano del Seguro Social IMSS (2020) Operational Definition of Suspected Case of Respiratory Viral Disease, Including COVID-*19* [Spanish]. Mexico City: Instituto Mexicano del Seguro Social (IMSS), p. 6.

[42] Williamson EJ (2020) Study protocol: comparison of different risk prediction modelling approaches for COVID-19 related death using the OpenSAFELY platform. Wellcome Open Research 5, 243. 10.12688/wellcomeopenres.16353.2 PMC11809169 39931522

