# Supplementary material for: Development and validation of the patient history COVID-19 (PH-Covid19) scoring system: a multivariable prediction model of death in Mexican patients with COVID-19
Source: Epidemiol Infect. 2020 Nov 26;148:e286. doi: 10.1017/S0950268820002903 (PMC7729170; doi:10.1017/S0950268820002903)
Supplement: Supplementary file 1 [file S0950268820002903sup.zip › S0950268820002903sup001.docx]

Epidemiology and Infection

Title: Development and Validation of the Patient History COVID-19 (PH-Covid19) Scoring System: A Multivariable Prediction Model of Death in Mexican Patients with COVID-19

Authors: J. Mancilla-Galindo, J. M. Vera-Zertuche, A. R. Navarro-Cruz, O. Segura-Badilla, G. Reyes-Velázquez, F. J. Tepepa-López, P. Aguilar-Alonso, J. de J. Vidal-Mayo, A. Kammar-García.

**Supplementary Material**

| Supplementary Table S1. Risk factors associated with death in Mexican patients with a positive diagnostic test for SARS-CoV-2 (model validation cohort). | | | | | |
| --- | --- | --- | --- | --- | --- |
|  | Regression coefficient | Standard error | HR (95%CI) | p value | Mean or proportion |
| Age | 0.057 | 0.001 | 1.05 (1.05-1.06) | <0.0001 | 43.87 |
| Sex (men) | 0.564 | 0.029 | 1.75 (1.66-1.86) | <0.0001 | 0.52 |
| Diabetes | 0.550 | 0.031 | 1.79 (1.63-1.84) | <0.0001 | 0.14 |
| COPD | 0.150 | 0.069 | 1.16 (1.01-1.33) | 0.03 | 0.01 |
| Immunosuppression | 0.469 | 0.091 | 1.59 (1.33-1.91) | <0.0001 | 0.01 |
| Hypertension | 0.200 | 0.032 | 1.22 (1.14-1.29) | <0.0001 | 0.17 |
| Obesity | 0.454 | 0.031 | 1.57 (1.48-1.67) | <0.0001 | 0.18 |
| CKD | 0.540 | 0.064 | 1.71 (1.51-1.94) | <0.0001 | 0.01 |
| HR: Hazard ratio, 95%CI: 95% Confidence Interval.  COPD: Chronic obstructive pulmonary disease, CKD: Chronic kidney disease | | | | | |
