# Supplementary material for: Development and validation of the patient history COVID-19 (PH-Covid19) scoring system: a multivariable prediction model of death in Mexican patients with COVID-19
Source: Epidemiol Infect. 2020 Nov 26;148:e286. doi: 10.1017/S0950268820002903 (PMC7729170; doi:10.1017/S0950268820002903)
Supplement: Supplementary file 1 [file S0950268820002903sup.zip › S0950268820002903sup003.docx]

Epidemiology and Infection

Title: Development and Validation of the Patient History COVID-19 (PH-Covid19) Scoring System: A Multivariable Prediction Model of Death in Mexican Patients with COVID-19

Authors: J. Mancilla-Galindo, J. M. Vera-Zertuche, A. R. Navarro-Cruz, O. Segura-Badilla, G. Reyes-Velázquez, F. J. Tepepa-López, P. Aguilar-Alonso, J. de J. Vidal-Mayo, A. Kammar-García.

**Supplementary Material**

| Supplementary Table S2. Risk of adverse events for each risk group in the PH-Covid19 scoring system. | | | | |
| --- | --- | --- | --- | --- |
| Model for Hospitalization | | | | |
| Score | Regression coefficient | Standard Error | OR (95%CI) | p value |
| -2 to 2 | Reference | | | |
| 3 to 5 | 0.57 | 0.03 | 1.77 (1.65-1.89) | <0.0001 |
| 6 to 8 | 1.39 | 0.03 | 3.99 (3.77-4.24) | <0.0001 |
| 9 to 15 | 2.54 | 0.03 | 12.7 (12.0-13.5) | <0.0001 |
| >15 | 3.42 | 0.08 | 30.5 (26.3-35.3) | <0.0001 |
| Model for Intubation | | | | |
| Score | Regression coefficient | Standard Error | OR (95%CI) | p value |
| -2 to 2 | Reference | | | |
| 3 to 5 | 0.72 | 0.12 | 2.05 (1.62-2.60) | <0.0001 |
| 6 to 8 | 1.67 | 0.11 | 5.31 (4.31-6.53) | <0.0001 |
| 9 to 15 | 2.81 | 0.10 | 16.67 (13.7-20.3) | <0.0001 |
| >15 | 3.29 | 0.15 | 28.97 (20.1-36.2) | <0.0001 |
| Model for Pneumonia | | | | |
| Score | Regression coefficient | Standard Error | OR (95%CI) | p value |
| -2 to 2 | Reference | | | |
| 3 to 5 | 0.67 | 0.04 | 1.94 (1.81-2.09) | <0.0001 |
| 6 to 8 | 1.43 | 0.03 | 4.19 (3.93-4.47) | <0.0001 |
| 9 to 15 | 2.56 | 0.03 | 12.9 (12.9-12.2) | <0.0001 |
| >15 | 3.28 | 0.07 | 26.64 (23.1-30.8) | <0.0001 |
| Model for Critical Care | | | | |
| Score | Regression coefficient | Standard Error | OR (95%CI) | p value |
| -2 to 2 | Reference | | | |
| 3 to 5 | 0.24 | 0.09 | 1.27 (1.04-1.54) | 0.02 |
| 6 to 8 | 1.14 | 0.08 | 3.14 (2.67-3.69) | <0.0001 |
| 9 to 15 | 2.16 | 0.07 | 8.63 (7.42-10.04) | <0.0001 |
| >15 | 2.84 | 0.13 | 17.12 (13.3-22.0) | <0.0001 |
| 95%CI: 95% confidence interval. | | | | |
