# Supplementary material for: Development and validation of the patient history COVID-19 (PH-Covid19) scoring system: a multivariable prediction model of death in Mexican patients with COVID-19
Source: Epidemiol Infect. 2020 Nov 26;148:e286. doi: 10.1017/S0950268820002903 (PMC7729170; doi:10.1017/S0950268820002903)
Supplement: Supplementary file 1 [file S0950268820002903sup.zip › S0950268820002903sup004.docx]

Epidemiology and Infection

Title: Development and Validation of the Patient History COVID-19 (PH-Covid19) Scoring System: A Multivariable Prediction Model of Death in Mexican Patients with COVID-19

Authors: J. Mancilla-Galindo, J. M. Vera-Zertuche, A. R. Navarro-Cruz, O. Segura-Badilla, G. Reyes-Velázquez, F. J. Tepepa-López, P. Aguilar-Alonso, J. de J. Vidal-Mayo, A. Kammar-García.

**Supplementary Material**

Supplementary Figure S1. Risk for adverse events in Mexican patients with a positive test for SARS-CoV-2, according to individual scores in the PH-Covid19 scoring system.


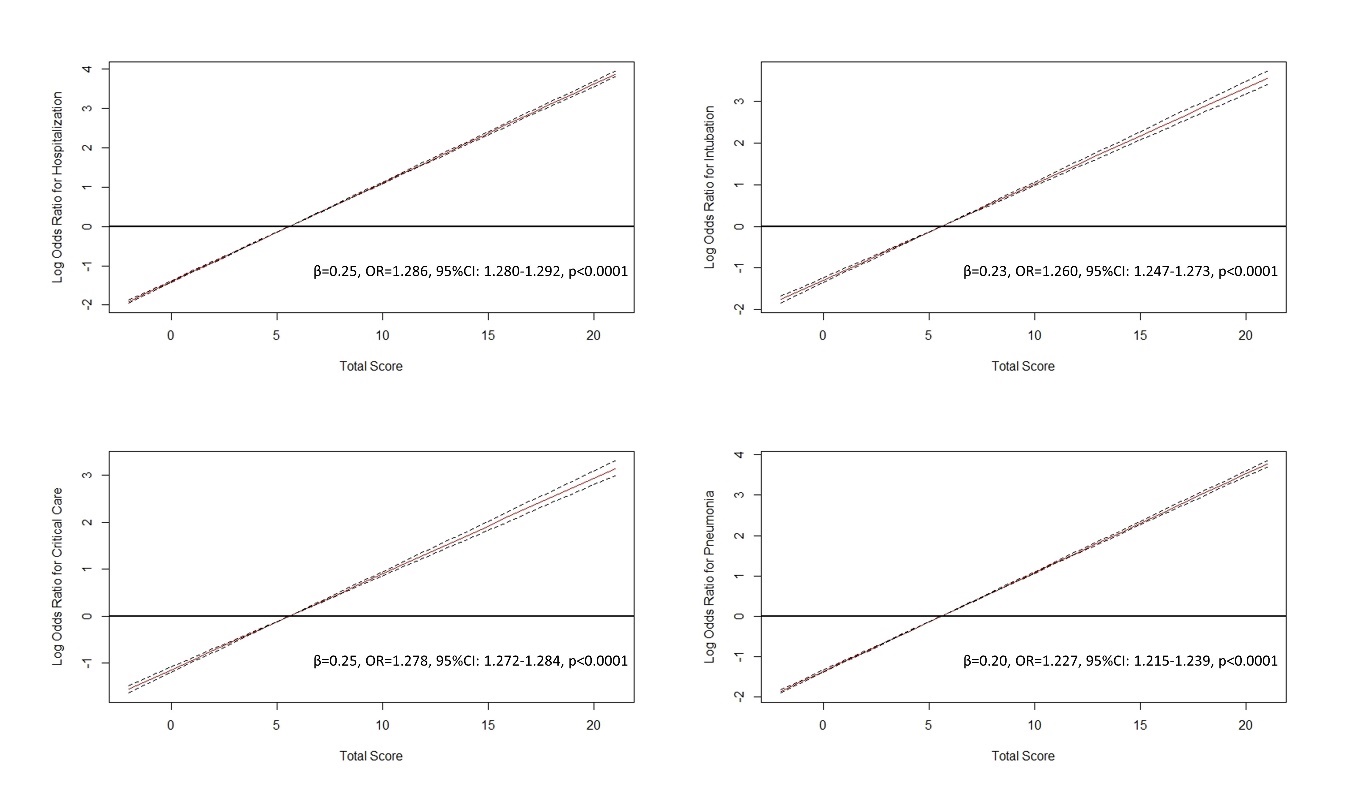


β: Regression coefficient, OR: Odds ratio, 95%CI: 95% coefficient interval. a) Risk of hospitalization, b) Risk of intubation, c) Risk of critical care, d) Risk of pneumonia.
